# Novel Triazine JPC-2067-B Inhibits *Toxoplasma gondii In Vitro* and *In Vivo*

**DOI:** 10.1371/journal.pntd.0000190

**Published:** 2008-03-05

**Authors:** Ernest J. Mui, Guy A. Schiehser, Wilbur K. Milhous, Honghue Hsu, Craig W. Roberts, Michael Kirisits, Stephen Muench, David Rice, J. P. Dubey, Joseph W. Fowble, Pradipsinh K. Rathod, Sherry F. Queener, Susan R. Liu, David P. Jacobus, Rima McLeod

**Affiliations:** 1 Department of Ophthalmology and Visual Science, University of Chicago, Chicago, Illinois, United States of America; 2 Jacobus Pharmaceutical Company, Inc., Princeton, New Jersey, United States of America; 3 Walter Reed Army Institute for Research, Silver Spring, Maryland, United States of America; 4 Department of Immunology, Strathclyde Institute for Biomedical Sciences, University of Strathclyde, Scotland, United Kingdom; 5 Krebs Institute for Biomolecular Research, Department of Molecular Biology and Biotechnology, The University of Sheffield, Sheffield, England, United Kingdom; 6 United States Department of Agriculture, Agricultural Research Services, Animal and Natural Resources Institute, Animal Parasitic Diseases Laboratory, Beltsville, Maryland, United States of America; 7 Department of Chemistry, University of Washington, Seattle, Washington, United States of America; 8 Department of Pharmacology and Toxicology, Indiana University School of Medicine, Indianapolis, Indiana, United States of America; 9 Department of Pediatrics, Committee on Molecular Medicines, Genetics, and Immunology and The College, University of Chicago, Chicago, Illinois, United States of America; McGill University, Canada

## Abstract

**Background and Methodology:**

*Toxoplasma gondii* causes substantial morbidity, mortality, and costs for healthcare in the developed and developing world. Current medicines are not well tolerated and cause hypersensitivity reactions. The dihydrotriazine JPC-2067-B (4, 6-diamino-1, 2-dihydro-2, 2-dimethyl-1-(3′(2-chloro-, 4-trifluoromethoxyphenoxy)propyloxy)-1, 3, 5-triazine), which inhibits dihydrofolate reductase (DHFR), is highly effective against *Plasmodium falciparum*, *Plasmodium vivax*, and apicomplexans related to *T. gondii*. JPC-2067-B is the primary metabolite of the orally active biguanide JPC-2056 1-(3′-(2-chloro-4-trifluoromethoxyphenyloxy)propyl oxy)- 5-isopropylbiguanide, which is being advanced to clinical trials for malaria. Efficacy of the prodrug JPC-2056 and the active metabolite JPC-2067-B against *T. gondii* and *T. gondii* DHFR as well as toxicity toward mammalian cells were tested.

**Principal Findings and Conclusions:**

Herein, we found that JPC-2067-B is highly effective against *T. gondii*. We demonstrate that JPC-2067-B inhibits *T. gondii* growth in culture (IC50 20 nM), inhibits the purified enzyme (IC50 6.5 nM), is more efficacious than pyrimethamine, and is cidal *in vitro*. JPC-2067-B administered parenterally and the orally administered pro-drug (JPC-2056) are also effective against *T. gondii* tachyzoites *in vivo*. A molecular model of *T. gondii* DHFR-TS complexed with JPC-2067-B was developed. We found that the three main parasite clonal types and isolates from South and Central America, the United States, Canada, China, and Sri Lanka have the same amino acid sequences preserving key binding sites for the triazine.

**Significance:**

JPC-2056/JPC-2067-B have potential to be more effective and possibly less toxic treatments for toxoplasmosis than currently available medicines.

## Author Summary

Toxoplasmosis is a neglected tropical disease, an emerging disease as well as a significant problem in developed countries causing a substantial health burden. Better medicines with less toxicity are greatly needed. Herein, we found that a novel triazine currently being advanced to clinical trials for malaria, JPC-2067-B, is highly effective against *T. gondii*. We demonstrate that JPC-2067-B inhibits *T. gondii* growth in culture (IC50 20 nM), inhibits the purified enzyme (IC50 6.5 nM), is more efficacious than pyrimethamine, and is cidal *in vitro*. JPC-2067-B administered parenterally and the orally administered pro-drug (JPC-2056) are also effective against *T. gondii* tachyzoites *in vivo*. A molecular model of *T. gondii* DHFR-TS complexed with JPC-2067-B was developed. We found that the three main parasite clonal types and isolates from South and Central America, the United States, Canada, China, and Sri Lanka have the same amino acid sequences preserving key binding sites for the triazine. Toxicology data are presented. JPC-2056/JPC-2067-B have potential to be more effective and less toxic treatments for toxoplasmosis than currently available medicines.

Toxoplasmosis is a neglected tropical disease as well as a significant illness affecting persons throughout the world and new and improved medicines are greatly needed for this and other apicomplexan infections [1]–[40]. In developing tropical countries, the problems for persons with AIDS can be exacerbated due to lack of both anti-retroviral treatment and anti-*Toxoplasma gondii* treatment. In this setting, this opportunistic pathogen causes substantial neurologic disease and treatment of this illness can be especially difficult because current gold standard medicines are unobtainable and/or unaffordable and, due to their toxicity, require monitoring which exceeds the capacity of many of the available health care systems. Toxoplasmic eye disease (chorioretinitis) is frequent in certain areas of Brazil and Colombia, areas where the gold standard drugs are particularly problematic, and is caused by atypical parasites that present major recrudescent and recurrent clinical problems. *T. gondii* is highly pathogenic and lethal in an emerging problem in French Guiana and Suriname [22],[34].

Throughout the world, new *T. gondii* infection during pregnancy can lead to devastating disease for the fetus and newborn infant, later impacting on the child's health and development and potentially on his/her later productivity [1]–[3]. In all areas of the world, this infection is life threatening and causes substantial neurologic damage for those with immune compromise. For some immunologically normal individuals this infection causes recurrent ophthalmologic and other organ damage [1]–[3].

Thus, toxoplasmosis is an important neglected disease in developing tropical countries, as well as an important cause of illness in developed countries in tropical and temperate climates [16]–[37]. All forms of toxoplasmosis (acute acquired, with or without symptoms; congenital; ocular; and in immune-compromised persons) occur throughout the world [1]–[3], [16]–[40]. In Europe and in the U.S. reports are that there are three predominant clonal types of *T. gondii* [22]–[24]. Clonal genetic type II *T. gondii* have been reported to predominate in France, Poland, and the U.S [24]. Atypical genetic types of *T. gondii* have been reported to occur in association with unusually severe eye disease in the U.S. in a small case series [25] and clonal type I parasites in some patients with AIDS and toxoplasmic encephalitis [26], but clonal type II parasites have been predominant among U.S. and European human isolates reported to date [24].

The presence of atypical *T. gondii* parasites in South and Central America have recently been discovered and found to be associated with significant human disease [27]–[32]. *T. gondii* strains in certain areas of Brazil, Colombia, and Guatemala [33] are atypical (rather than the European and U.S. predominant three clonal types) and are often genetically polymorphic [34]. In the Minas Girais area of Brazil (36), infection with *T. gondii* is common. In Erechim, Rio Grande do Sul 17.7% of the population had ocular toxoplasmosis. In Colombia, where atypical, non clonal type I, II, or III, parasites are endemic, frequency of retinal lesions of ocular toxoplasmosis in medical residents was 6% [31]. Severe congenital disease occurs in 0.5% of live births in Colombia [32]. In addition, in sharp contrast to clonal type II parasites predominating in Europe, in certain tropical countries with wild felids and a wide variety of wild mammals, the parasites are genetically more diverse and the many potential mammalian hosts apparently appear to be associated with the presence of greater genetic diversity in these atypical strains [34]. For example, in French Guiana [34], parasites have many felid hosts and these parasites have been considered to be representative of those found in the tropical Amazon reservoir [35]. In Northern Coastal South America they have caused lethal and severe diseases in humans including French soldiers; these diseases have included persistent neurologic findings, Guillain Barre syndrome, severe pneumonia, and death [37].

Substantial waterborne epidemics have occurred in Brazil [38], in Canada [39], and in U.S. soldiers in Panama [40], considered to be secondary to feral or wild cats in proximity to drinking water [38]–[40]. A cat excretes up to 20 million unsporulated oocysts during just a few days, these become infections following sporulation, and even 1 oocyst is infectious [1]. Oocysts excreted by cats can persist in warm moist soil for up to a year and in seawater for up to 6 months. Thus infection is readily spread in nature and is a very common infection throughout the world.

Congenital toxoplasmosis is a significant problem in the developed and developing world, but it is particularly difficult in parts of the developing world to obtain the gold standard medicines, pyrimethamine and sulfadiazine. For example, in Colombia, it is not possible to obtain pyrimethamine and sulfadiazine to treat congenitally infected infants. The cost of compounding, administering and monitoring the safe use of these medicines also would likely be prohibitive for most residents of rural Africa or Central and South America.

These issues also are especially problematic for those with AIDS and toxoplasmosis in the developing world, because the medicines and the monitoring required for their proper and safe use are often also both unavailable and unaffordable [17]. In patients with AIDS, toxoplasmosis is a major, presenting, opportunistic, central nervous system infection and this is also the case throughout the entire course of the AIDS infection when HAART is not obtainable or affordable [17]. Early in the AIDS epidemic in the U.S. and Europe, approximately half of seropositive, i.e., individuals with chronic *T. gondii* infection, and AIDS infected individuals developed toxoplasmic encephalitis [16]. An example of the likely magnitude of this problem can be seen when one considers sub-Saharan Africa [17]. In sub-Saharan Africa approximately 25 million people have HIV infection/AIDS [18], and co-infection with *T. gondii* frequently remains undetected and thus untreated [17]. *T. gondii* seroprevalence ranges from 35% to 84% in different African countries south of Sahara (reviewed in [19]). Because approximately 30–50% of persons who have been co-infected with HIV and *T. gondii* in the U.S. or Europe in the pre-HAART era ultimately developed toxoplasmosis, the high seroprevalence in sub-Saharan Africa combined with the HIV-pandemic indicate that 2.5–10 million people in this region are likely to be at risk of dying from toxoplasmosis. A recent study of types of parasites using a SAG2 marker indicated that all three SAG2-types have been found in chickens in Africa [20].

HIV infection is not the only immunodepressive health condition that is frequent in the developing world and that worsens manifestations of toxoplasmosis. In India, adults who were malnourished but otherwise immunologically normal and who were without HIV infection had severe, symptomatic toxoplasmic encephalitis [21].

Ideal medicines to treat toxoplasmosis in developing tropical countries would be effective, easily obtained and affordable, without toxicity, including hypersensitivity and neutropenia which requires co-adminstration of leukovorin and careful monitoring of neutrophil count. They also would be non teratogenic so the fetus and pregnant woman could be treated. In addition they would be rapidly effective, safe, without any toxicity, when available in pediatric suspensions. Further, they would be available parenterally for those who are acutely ill and unable to take oral medicines. They would be effective against all isolates of *T. gondii* (all three clonal types and atypical parasites). Ideally a medicine would also be cidal against bradyzoites. An ideal medicine for toxoplasmosis would have superb penetration into the eye and brain. These would be major advantages for this neglected disease throughout the world, and especially important in developing countries. JPC-2056/JPC-2067-B have the potential to address some of these issues (e.g. cidal for tachyzoites, less toxicity, available for oral and parenteral use, and potentially available in pediatric suspensions that are stable without refrigeration). Further testing and development will reveal whether JPC-2056/JPC-2067-B can address the other characteristics of an ideal anti-toxoplasmosis medicine.

Current treatment of toxoplasmosis includes the combination of a folic acid antagonist and an inhibitor of dihydropteroic acid synthesis: The gold standard treatment has been the classic anti-malarial combination of pyrimethamine and sulfadiazine. *In vitro* and *in vivo* experimental models of toxoplasmosis parallel this clinical approach [1],[3]. Herein, results using those same *in vitro* and *in vivo* toxoplasmosis models with a new anti-malarial candidate, JPC-2067B (4, 6-diamino-1, 2-dihydro-2, 2-dimethyl-1-(3′-(2-chloro-4-trifluoromethoxyphenoxy) propyloxy)-1, 3, 5-triazine) and its pro-drug JPC-2056 are presented [4]–[10]. This new anti-malarial class [4]–[10], without a sulfonamide, has dramatic potency against multi-drug resistant *Plasmodium falciparum* strains [4]–[10].

We have waited a long time for a representative of this series of compounds to advance to the clinic for the treatment of *T. gondii* infection. This is especially important for those with this infection who are immune compromised and potentially also for those infected in pregnancy and *in utero*. New medications are needed because the classic gold standard medications have substantial toxicity [1],[2]. Moreover, pyrimethamine cannot be used in the first trimester of pregnancy, as folate depletion is detrimental to fetal development [1]. Neutropenia is a common toxicity with pyrimethamine treatment even when leukovorin is administered in conjunction with this medicine [3]. Furthermore, pyrimethamine is generally administered in a synergistic combination with sulfadiazine which has substantial associated hypersensitivity [2] and toxicity (e.g. kidney stones or hepatic or renal complications). New medicines are greatly needed for individuals suffering from toxoplasmosis

**Figure 1:**
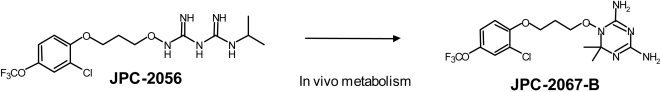
Structures of triazines JPC-2056 and JPC-2067-B.

The extremely promising candidate, JPC-2067-B, comes from a pre-clinical anti-malarial series well known in malariology by the name of the related metabolite WR99210 (4,6-diamino-1,2-dihydro-2,2-dimethyl-1-[3′(2,4, 5-trichlorophenoxy)propyloxy]-1, 3, 5-triazine) [4]–[10]. *In vitro* anti-malarial testing of WR99210 against drug-sensitive and drug-resistant strains has shown high potency and full activity against *P. falciparum* strains not responsive to pyrimethamine, proguanil or chloroquine with an ED_50_ of 0.05 ng/mL *in vitro*. As yet there is no strain resistant to this class of compounds. WR99210 is discussed here in order to provide a common point of cross reference. Like proguanil, the new clinical candidate JPC-2056 (Figure 1) is a biguanide pro-drug which is metabolized *in vivo* to the active dihydrotriazine JPC-2067-B (Figure 1). For *in vitro* testing the metabolite must be used; for oral usage the biguanide must be given. The ongoing work in development and progression to use in the care of patients of this very promising anti-malarial clinical candidate (Jacobus *et al* , unpublished) also is useful in development of the same medicine for treatment of toxoplasmosis.

The previously described triazine WR99210 and its pro-drug, PS-15, were developed in response to resistance of *P. falciparum* to pyrimethamine and cycloguanil [4]–[11]. WR99210 was found to be a very tight binding and potent inhibitor of *P. falciparum* DHFR-TS [4]–[11]. WR99210 and PS-15 also were highly active *in vivo* against *P. falciparum*, with activity 2 logs greater than that of pyrimethamine. These compounds were also highly active against *P. vivax*, without cross-resistance to other antifolates (S. Hunt, personal communication). The therapeutic/toxic ratio is increased because the high avidity of these compounds for the *P. falciparum* DHFR differs from its lower avidity to mammalian DHFR [11]. Unfortunately, toxicity of WR99210 limited its development and use and it will not be a clinically useful compound.

We previously evaluated the active triazine metabolite of proguanil (cycloguanil) against *T. gondii* tachyzoites [12], and more recently found that WR99210 was also highly active against *T. gondii in vitro* and *in vivo* when administered parenterally [13]. PS15 also was found to be effective *in vivo* [13].

A major drug discovery effort over the past 6 years has identified an analog of WR99210, JPC-2067-B, which has superior pharmacological characteristics. Importantly, pro-drug JPC-2056, is easily absorbed, bioavailable, and relatively nontoxic. In studies with *P. falciparum*, oral administration of JPC-2056 resulted in conversion to the JPC-2067-B which was cidal for the malaria parasite. The high potency and selectivity of JPC-2067-B for inhibition of apicomplexan parasite DHFR relative to mammalian DHFR reduces the likelihood of neutropenia, thus enhancing the margin of safety and convenience in monitoring white blood counts with its use. JPC-2056 was also as active as monotherapy *in vitro* as the synergistic combination of pyrimethamine and sulfadiazine and is currently being advanced to clinical trials, leading to a new and markedly improved class of anti-folate medicines for the treatment of malaria.

The effect of JPC-2067-B on *T. gondii* is of considerable interest and importance. The lack of toxicity of JPC-2067-B and the favorable absorption and distribution profile of its prodrug JPC-2056 offers the possibility of overcoming the limitations of pyrimethamine. The benefit of greater specificity for the parasite rather than host DHFR could have the dual advantage of reducing host toxicity while eliminating the need for simultaneous administration of a sulfonamide. Whether an IC 50 of 6.5 nM is sufficient to be used as a single agent for either malaria or toxoplasmosis or would be better used in conjunction with another anti-microbial *in vivo* under clinical conditions remains to be determined.

Structures of JPC-2067-B and its corresponding pro-drug JPC-2056 (Jacobus Pharmaceutical Company, Princeton, NJ) are shown in Figure 1. The biguanide pro-drug is converted *in vivo* to the biologically active dihydrotriazine through P450 metabolism in the liver, and so *in vitro* experiments are always conducted with the dihydrotriazine (JPC-2067-B). The overall aim of the experiments was to determine effect of the dihydrotriazine on *T. gondii in vitro* and *in vivo* and inhibitory effect of the dihydrotriazine on *T. gondii* that was observed is described herein.

## Methods

### Parasites and assessment of effect of inhibitors on *T. gondii* tachyzoites in tissue culture and cells in tissue culture

Tachyzoites of the RH strain of T. gondii were passaged in human foreskin fibroblasts (HFF). They were used to infect fibroblasts to determine antimicrobial effects of candidate compounds. Outcome was assessed with microscopy and uracil uptake after four days in culture as described [8],[12],[13]. Briefly, for testing of inhibitors *in vitro* against *T. gondii* tachyzoites, four-day old confluent cultures of human foreskin fibroblasts (HFF) were infected with 10^3^ tachyzoites and cultured for 1 hour to allow parasite invasion. Inhibitor was added and cells cultured for 3 days. They were supplemented with ^3^H uracil and incubation extended for a further day, whereupon uracil incorporation into cells and thus parasite growth were assessed by liquid scintillation counting [8],[12],[13]. Studies were performed with inhibitors as described in [8],[12],[13]. Lack of toxicity for mammalian host cells was demonstrated first by visual inspection of the monolayer and by parallel concomittant evaluation of separate ^3^H thymidine incorporation assays by non-confluent HFF cell monolayers.

### JPC-2067-B for use in *in vitro* (tissue culture) and *in vivo* studies

For *in vitro* studies, a stock solution of JPC-2067-B was initially dissolved in 100% dimethyl sulfoxide (DMSO) and then diluted in complete tissue culture medium (IMDM-C) [IMDM with NaHCO_3_ and 25 mM Hepes (Cambrex Bio Science, Walkersville, MD), 10% fetal bovine serum (Gibco, Grand Island, N.Y.), 1× antibiotic-antimycotic solution (Cellgro, Mediatech), and 2 mM L-glutamine (Gibco). Working concentrations of JPC-2067-B were made using IMDM-C. Concentrations measured ranged from 10 to 100 nM. For certain *in vivo* studies, JPC-2067-B was initially dissolved in 100% DMSO and then diluted 100 fold in 1× PBS without calcium or magnesium (Cellgro) and administered intra-peritoneally (i.p.) 15 minutes following i.p. inoculation of the parasite. In other *in vivo* studies, the orally bioavailable pro-drug JPC-2056 (40 mg/kg/dose, bid) was administered per orally by gavage beginning one day following i.p. inoculation of the parasite.

### DHFR enzyme activity and its inhibition by JPC-2067-B: Effect against *T. gondii* DHFR compared to *Pneumocystis carinii*, *Mycobacterium avium-intracellulare* and rat liver DHFRs

DHFR from *Pneumocystis carinii* was produced as the recombinant enzyme expressed in *Escherichia coli* [41]. The sequence of the protein was identical to that predicted for the previously reported gene sequence [4]. DHFR from *T. gondii* was isolated directly from RH strain *T. gondii* grown in culture on Chinese hamster ovary cells lacking DHFR (CHO/dhfr^-^, American Type Culture Collection 3952 CL) [42]. Organisms were introduced into a confluent monolayer and harvested when the mammalian cells were lysed. The 100,000× g supernate was stored in liquid nitrogen.

*Mycobacterium avium-intracellulare* used in these studies was a clinical isolate (serovar 4) from Indiana University School of Medicine, Department of Pathology. The strain was maintained on Lowenstein-Jensen slants (Baxter Scientific) grown at room temperature. To produce enzyme, the organism was grown in Middlebrook 7H-9 liquid medium at 37°C to an OD_660_ of 0.5 to 0.7, which took several weeks. At harvest, the bacteria were centrifuged, sonicated, and the 100,000 Xg supernate was stored under liquid nitrogen until assay. These supernates contained both DHFR and dihydroopteroate synthetase activity.

Rat liver DHFR was prepared from livers of female Sprague-Dawley rats. The 100,000× g supernate was partially purified by ammonium sulfate precipitation; the 50–90% precipitate was re-dissolved and stored in liquid nitrogen.

The spectrophotometric assay for DHFR was optimized for temperature and concentration of substrate and cofactor for each enzyme. The standard assay contained Na phosphate buffer pH 7.4 (40.7 mM), 2-mercaptoethanol (8.9 mM), NADPH (0.117 mM), dihydrofolic acid (0.09 mM), KCL (150 mM), and sufficient enzyme to produce a change in OD_340_ of 0.035/minute at 37°C. The reaction was continuously recorded for 3 minutes. Activity under these conditions was linear with enzyme concentration over a 4-fold range. The low background activity in the absence of dihydrofolic acid was subtracted from all rates.

DHFR was assayed with several concentrations of inhibitor to produce rates ranging from 1 to 90% of the uninhibited rate. At least three concentrations were required for calculation; most curves contained five concentrations. Semi-logarithmic plots of the data gave sigmoidal curves that were fit by non-linear methods to determine the concentration yielding 50% inhibition (IC50) [Prism 4.0 (GraphPad)].

### Effect against *T. gondii* DHFR compared with *P. falciparum* DHFR and human DHFR

DHFR from *T. gondii* [43] prepared as above was also directly compared to purified recombinant *P. falciparum* DHFR (pfDHFR) and purified recombinant human DHFR (hDHFR). The hDHFR was from pDFR plasmid [10]. The enzyme was purified following ammonium sulfate precipitation, methotrexate:agarose affinity chromatography, and finally a Superdex 200 size exclusion column. The pfDHFR isolation methods were those reported previously [11],[44]. Pyrimethamine and JPC-2067-B were tested for activity against recombinant pfDHFR, recombinant hDHFR and the *T. gondii* lysate DHFR. The same buffer as used in the other assays comparing *T. gondii* DHFR with DHFRs from rat liver *P. carinii* and *M. avium intracellulare* was used but the maximal activity, temperature, and length of observation were adjusted for assays on the specific plate reader. The series of pfDHFR and hDHFR assays were run twice for hDFHR and three times for pfDHFR and the representative data are shown (see Results). The tgDHFR sample was exhausted after one set of assays at a lower activity than the others (uninhibited change in OD_340_ of 0.004/min versus 0.02/min for the recombinant enzymes). The reaction was setup at 23°C, the plate loaded, and the OD_340_ recorded at 20 second intervals for 10 minutes. The first 8 minutes were used to generate linear fit slopes in Excel. Each concentration has been reported as the mean of 5 replicate reactions with the standard deviation reported as the error. Results are expressed as the percent of control activity versus log concentration of inhibitor. Prism 5.0 was used to generate curves from 12 different concentrations of inhibitor using a non-linear fit method.

### Quantitation of JPC-2067-B

JPC-2067-B levels were quantitated using an HPLC system comprised of a Spectra System P4000 pump, AS300 autosampler, UV2000 detector and a ChromJet integrator. The column is a Phenomenex Synergi 4μ MAX-RP 80A 150×4.6 mm, s/n 219259. Elution was effected with a gradient of Mobile Phase A (0.05% aqueous TFA) and Mobile Phase B (0.025% TFA in acetonitrile). The flow rate was 0.5 ml/min, the injection volume was 20 µl and the detector was set to 290 nm. Observed retention times for WR99210, PS-15 and JPC-2067-B were 9.5, 15.7 and 9.1 minutes, respectively.

### *T. gondii* Infection of mice

Tachyzoites also were used to infect mice. Outbred Swiss Webster mice were bred in our specific pathogen free colony. When they were approximately 30 g, they received 10,000 RH strain tachyzoites intra-peritoneally (i.p.); numbers of parasites present in peritoneal fluid were counted four days later as described [8],[13]. Mice were maintained and utilized in accordance with IACUC and NIH guidelines and approvals.

### Studies of effect of peritoneal administration of JPC-2067-B on murine toxoplasmosis

JPC-2067-B was administered parenterally. In initial studies, this was given 15 minutes after i.p. infection and then each day for four days(1.25 mg/kg/day). Peritoneal parasite burden was quantitated on the fourth day after injection. Control mice received 1% DMSO in PBS.

### Studies of effect of oral administration of JPC-2056 on murine toxoplasmosis

Beginning one day following infection of outbred SW mice with tachyzoites of the RH strain of *T. gondii* mice received JPC-2056 by gavage at a concentration of 40 mg/kg in 0.5 ml twice daily. Peritoneal *T. gondii* burden was determined on day 4 following infection.

### PCR and bioinformatics of DHFR in various clonal and atypical strains of *T. gondii*

Sequences of DHFR in each of the conventional parasite clonal types (RH, type I; Me49 type II; and VEG, type III) from the data base and by PCR using strains (isolates) from Brazil, Guyana, Guatemala, Canada, China, and Sri Lanka [45]–[50] were determined with PCR using cDNA or g DNA as template. The primers used were: Forward, 5′-AGGGACGGTGAAGTTTCGCTTTA-3′; Reverse, 5′-TTTCCGGTCTTCTTCGTCCATCCA-3′.

### Modeling of DHFR

Modeling of the *T. gondii* DHFR was based upon the crystal structure of the closely related *P. falciparum* DHFR in complex with WR99210, NADPH and dUMP (pdb id 1j3i), using the structure based sequence alignment as a guide. Those residues which displayed sequence variation between *P. falciparum* and *T. gondii* DHFR and were located within 4Å of the ligand binding pocket were analysed to look for significant differences.

### Toxicology studies

A 42-day toxicology study in CD-1 mice at doses up to 98 mg/kg evaluating well-being, weight gain, and histopathology was performed. A comparable 42-day toxicology study in *Macaca fascicularis* also was performed. 7.5 mg/kg was established as the NOAEL (No Observed Adverse Effect Level). JPC-2056 and JPC-2067 were assayed in the Ames Test with and without microsomal activation with tester strains TA97, TA98, TA100, TA102 and TA1535.

### Statistical analysis

Significance of differences was determined using a Mann Whitney U test or Student's T-test. All experiments were performed at least twice and representative experiments are shown.

## Results

### Effect of JPC-2067-B on *T. gondii* tachyzoites *in vitro*

**Figure 2:**
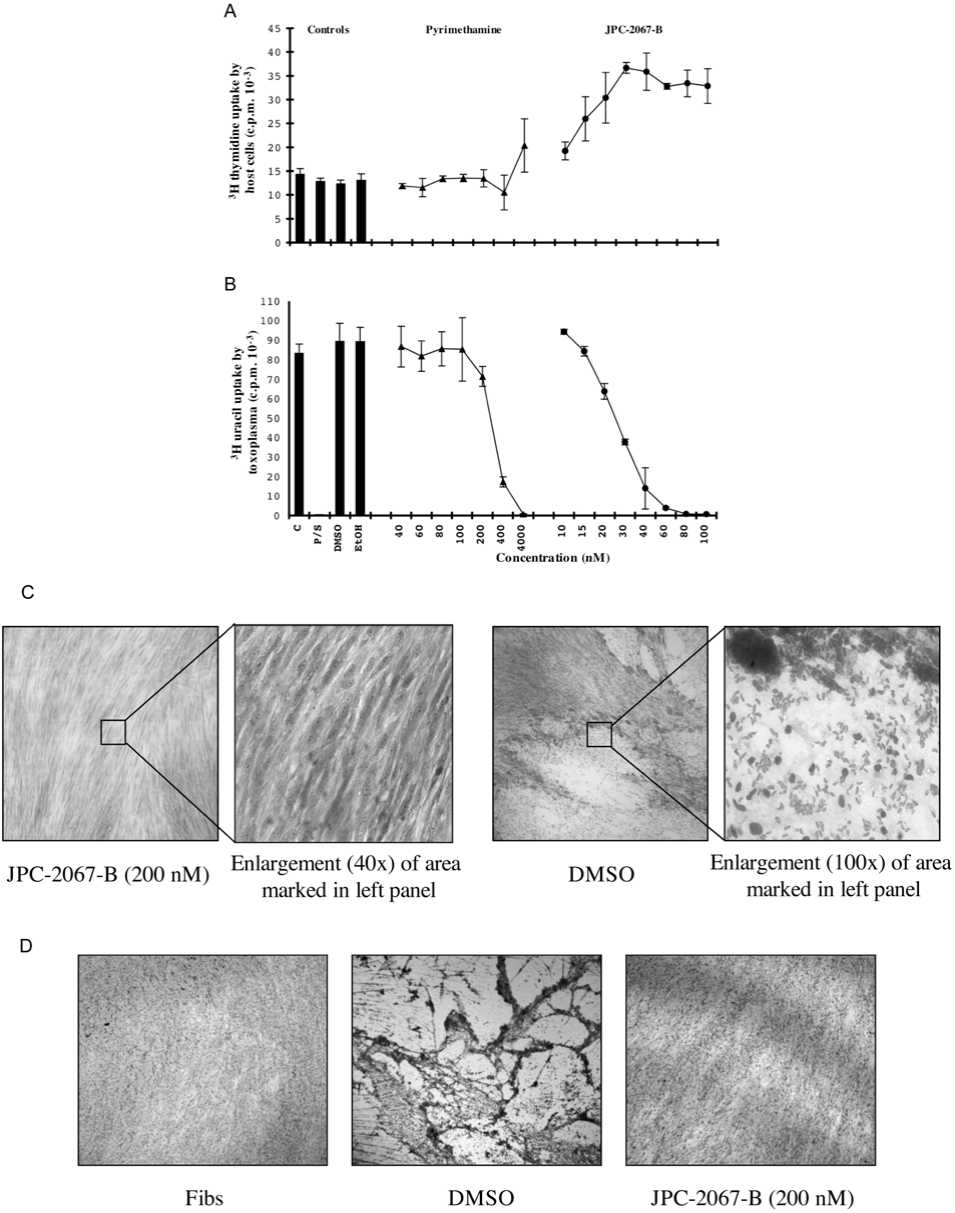
Effect of JPC-2067-B on *T. gondii* in human foreskin fibroblasts. A. Thymidine uptake assay demonstrates no toxic effect on host cells. B. Uracil uptake assay demonstrates that JPC-2067-B is effective against *T. gondii* at low nanomolar concentrations. C. Micrographs showing marked inhibition of *T. gondii* by JPC-2067-B. Note absence of plaques and parasites in treated cultures. Concentrations prepared are shown. D. Micrograph showing absence of destruction of monolayers infected, exposed to JPC-2067-B for 4 days and cultured for prolonged times with *T. gondii*. The control monolayer was completely destroyed by 5 days of culture. This contrasts with similarly infected monolayers in the micrograph exposed to JPC-2067-B for 4 days and then with the JPC-2067-B removed. No plaques or *Toxoplasma* were seen throughout 52 days of culture, demonstrating that the JPC-2067-B is cidal and no drug resistant mutants were selected in this experiment. No plaques were present from 4 days through the subsequent 1 and 1/2 months after *in vitro* challenge.

JPC-2067-B was highly effective against *T. gondii* tachyzoites in tissue culture. A representative experiment of two trials is shown in Figures 2A and B. The IC50 and IC90 for JPC-2067-B were ≈20 nM and 50 nM, respectively. Differences between control and treated groups at these and higher concentrations were statistically significant (p<0.05). We observed some precipitation of the compounds in the stock solutions, and so the supernatant was analyzed by HPLC. We found that the actual concentrations measured in the supernatants were approximately four-fold less than the initial amounts. The actual amounts measured are shown in Figure 2. Data are shown both as uptake of ^3^H uracil into nucleic acid of the parasites (Figure 2B) and with micrographs of Giemsa stained microscopic preparations (Figure 2C), with efficacy confirmed by both methods. Direct comparison of WR99210 and JPC-2067-B and similar IC 50 and 90s for WR99210.

### Lack of toxicity of JPC-2067-B for human fibroblasts tested concomitantly with *T. gondii* tachyzoites

Human foreskin fibroblasts were tested concomitantly with *T. gondii* tachyzoites with increasing concentrations of JPC-2067-B. Data from a representative experiment are also shown in Figure 2A and demonstrate no toxicity measured as uptaked of tritiated thymidine by nonconfluent fibroblasts. The increased uptake of thymidine in these cultures remains unexplained but also has been noted with certain other compounds such as triclosan.

### JPC-2067-B is cidal for *T. gondii*

In separate experiments, to determine whether JPC-2067-B would be cidal for *T. gondii*, cultures were maintained for 52 days after removing JPC-2067-B on the 4^th^ day of culture. No plaques or growth of parasites were detected (Figure 2D). The absence of growth following removal of JPC-2067-B from HFF exposed to *T. gondii* indicates that this compound is “cidal” and not merely “static” for *T. gondii*.

### *In vivo* effect of parenteral administration of JPC-2067-B and oral administration of the pro-drug JPC-2056 on toxoplasmosis

**Figure 3:**
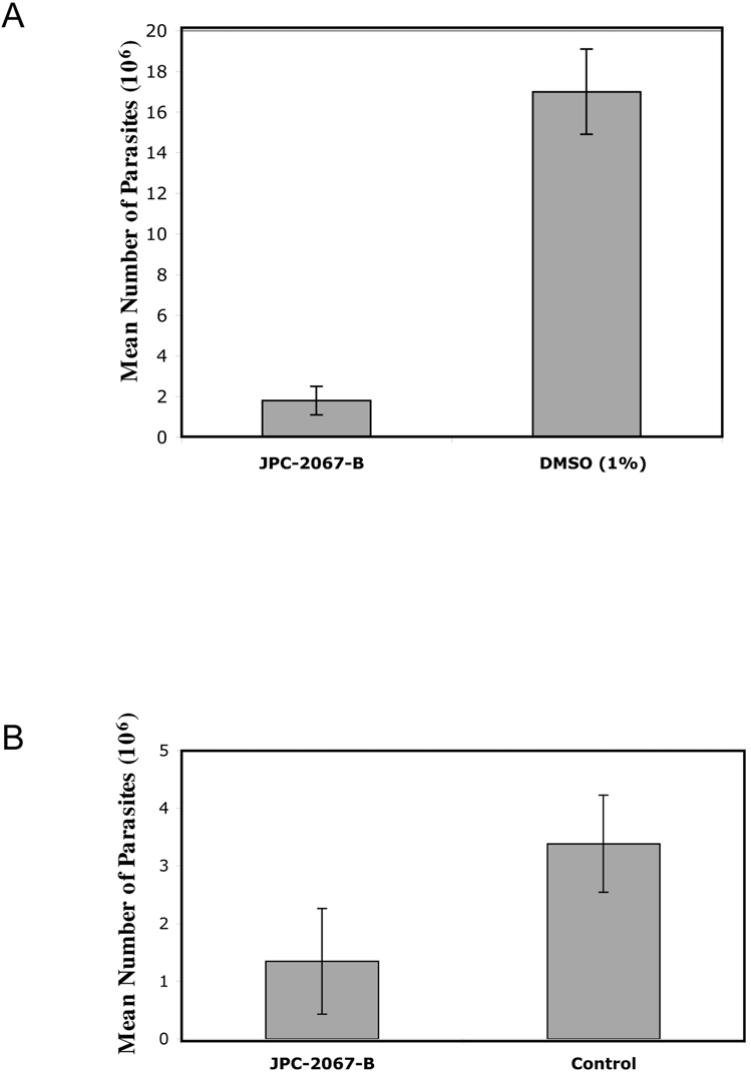
Reduction of numbers of parasites in peritoneal fluid. A. Reduction of numbers by i.p. treatment of mice with JPC-2067-B. B. Reduction of numbers of parasites following treatment of mice with the pro-drug JPC-2056. JPC-2056 was administered by gavage. JPC-2056 is converted into the active compound, JPC-2067-B.

JPC-2067-B was also highly effective against *T. gondii* tachyzoites in a mouse model. A representative experiment with JPC-2067-B is shown in Figure 3A. In the experiment in Figure 3A, mice were infected i.p. with 10,000 tachyzoites of the RH strain of *T. gondii* for 15 minutes prior to initial treatment with JPC-2067-B.

For these parenterally treated mice, female mice received a dose of 1.25 mg/kg/day of JPC-2067-B, administered i.p. for the next 3 days. Control mice received an equivalent amount of DMSO (1%) in 1× PBS. In a separate experiment, DMSO at this concentration was shown not to modify subsequent parasite numbers when compared with i.p. inoculation of PBS. Mice treated with JPC-2067-B appeared sleek and active 4 days after infection. In contrast, infected control mice appeared ill, with ruffled fur and hunched posture. Intraperitoneal parasite numbers were reduced by two logs with treatment with JPC-2067-B on the fifth day after injection of parasites (Figure 3A). These differences between control and treated mice were statistically significant (p<0.05).

In addition, a similar experiment was performed with oral administration of the orally bioavailable pro-drug JPC-2056 (40 mg/kg/dose, bid) beginning one day following i.p. inoculation of the parasite. Parasite number in peritoneal fluid was quantitated three days after that, i.e. the fourth day following infection. For the mice orally treated with JPC-2056 there were similar significant differences in parasite peritoneal burden on the third day of treatment (Figure 3B, p<0.03).

### Effect of JPC-2067-B on *T. gondii* DHFR enzyme activity compared with effects on DHFR enzyme activity from mammalian cells, *Pneumocystis carinii*, and *Mycobacterium avium-intracellulare*

**Figure 4:**
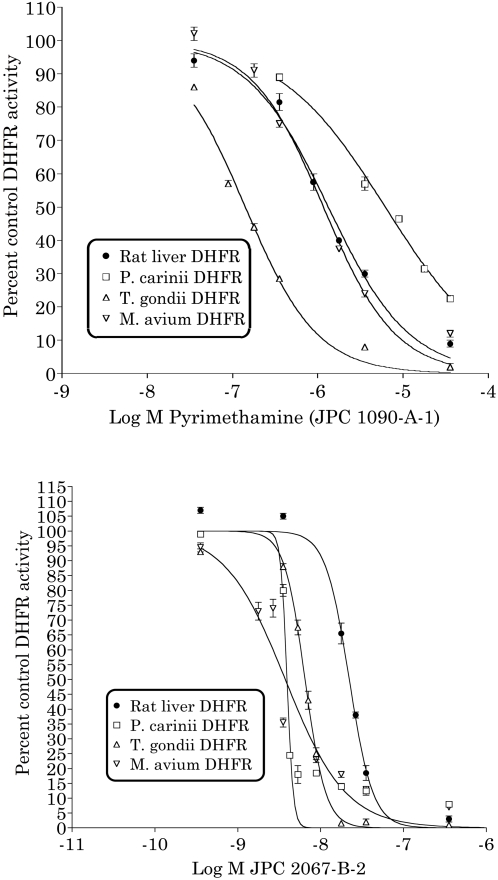
Activity of pyrimethamine and JPC-2067-B against *T. gondii* and rat liver DHFRs and DHFRs of other opportunistic pathogens.

The IC50 values determined for reference compound pyrimethamine (JPC-1090) were in agreement with prior assays of the compound (S. Queener, unpublished data). Both JPC-1090 and JPC-2013 (cycloguanil) had IC50 values in the micromolar range and were not significantly selective for pathogen DHFR (Table 1). JPC-208 (WR92210) was more potent, with IC50 values in the nanomolar range, but was not selective. JPC-2067-B had nanomolar IC50 values for the DHFRs from all three pathogens and higher IC50 value for the mammalian DHFR, yielding about 3.4 to 5.9 fold selectivity. The potency for this compound greatly exceeds the concentration of pyrimethamine used clinically (Figure 4).

**Table 1:** Comparison of IC50 of JPC-2067-B against DHFRs of *T. gondii* and other opportunistic pathogens that are harmful to patients with AIDS.

Compound	Rat liver DHFR IC50 (µM)	Pc DHFR IC50 (µM)	Tg DHFR IC50 (µM)	Mav DHFR IC50 (µM)
JPC-1090-A-1 (Pyrimethamine)	1.32 (1.14–1.55)	6.17 (5.45–6.98)	0.14 (0.12–0.16)	1.2 (0.93–1.45)
JPC-2013-B-1	1.73 (1.48–2.03)	8.9 (8.2–9.7)	0.41 (0.38–0.43)	12.1 (11.2–13.1)
JPC-208-B (WR99210)	0.00081 (0.0007–0.00093)	0.000265 (0.00025–0.00029)	0.000602(0.00057–0.00063)	0.00057 (0.00047–0.00071)
JPC-2067-B	0.0222 (0.0206–0.0238)	0.00393 (0.00371–0.00417)	0.0065 (0.00626–0.00674)	0.00375 (0.00291–0.00483)

Values shown are micromolar IC_50_ values (95% confidence limits).

Semilogarithmic plots of the data yielded normal sigmoidal curves for pyrimethamine and cycloguanil (Hill slope of the normalized log-concentration-response curve was about −1) but both WR-99210 and JPC-2067-B yielded very steep curves for the DHFRs from rat liver, *P. carinii*, and *T. gondii*; these compounds produced normal dose response curves with *M. avium* DHFR. The steep Hill slopes for JPC-208 and JPC-2067-B suggests that the interaction of these compounds with these enzymes is not following a simple 1∶1 interaction expected with a competitive inhibitor.

### Effect of JPC-2067-B on *T. gondii* DHFR enzyme activity compared with effect on *P. falciparum* and human DHFR enzyme activities

**Figure 5:**
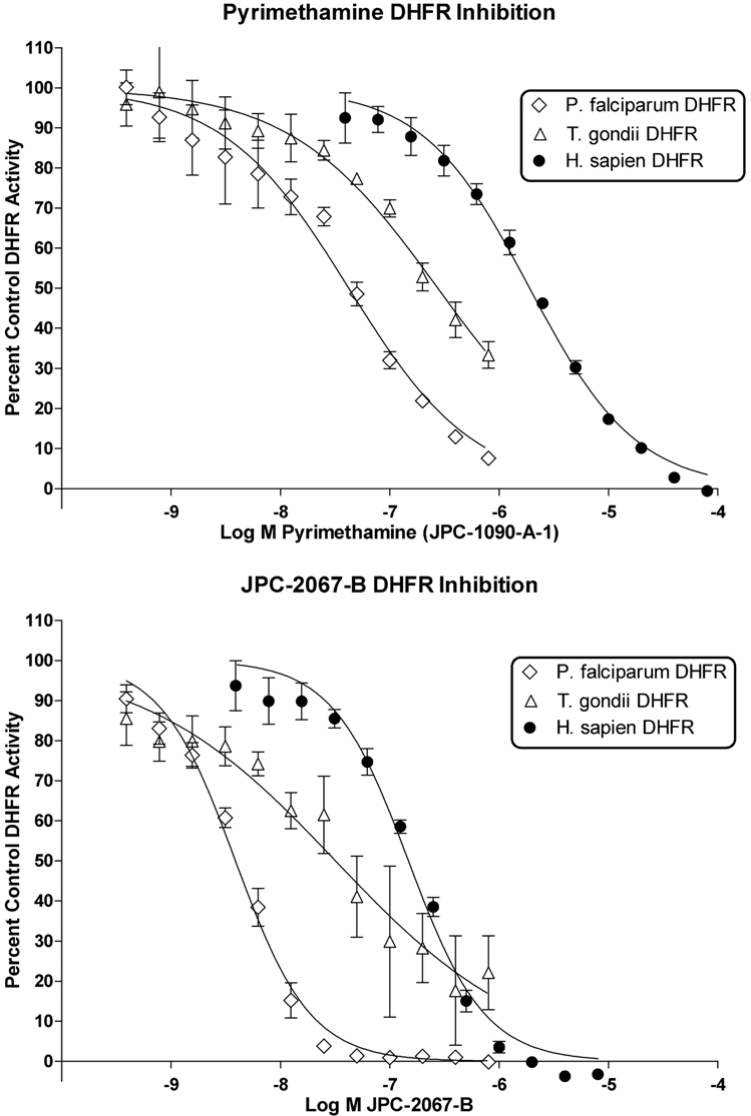
Activity of pyrimethamine and JPC-2067-B against *T. gondii*, *P. falciparum*, and human DHFRs.

In Figure 5 and Table 2, the IC_50_ for *P. falciparum* DHFR was 3.9 nM, *T. gondii* DHFR was 32 nM, and human DHFR was 150 nM. For pyrimethamine, the IC_50 _for *P. falciparum* was 42 nM, for *T. gondii* DHFR was 280 nM, and for human DHFR was 1,900 nM.

**Table 2:** Comparison of IC_50_ of JPC-2067-B and Pyrimethamine against recombinant PfDHFR and hDHFR and DHFR activity of Tg lysates.

Compound	hDHFR IC_50_ (µM)	PfDHFR IC_50_ (µM)	TgDHFR IC_50_ (µM)
JPC-1090-A-1 (Pyrimethamine)	1.90 (1.77–2.05)	0.0422 (0.0369–0.0483)	0.275 (0.236–0.321)
JPC-2067-B	0.148 (0.136–0.162)	0.00388 (0.00364–0.00414)	0.0320 (0.0245–0.0417)

Values shown are micromolar IC_50_ values (95% Confidence Intervals).

The differences in values between Figures 4 and 5 may be due to variations in assays. Assays towards the comparison to inhibition of opportunistic pathogens are run on partially purified lysates at 37°C for a shorter duration while this set of assays is run in a high-throughput manner with several recombinant enzymes and a more drawn out observation time at 23°C. The amount of enzyme used has been reduced to extend the length of observation and minimize the effect of data points lost during plate setup. These differences in methodology likely explain the slight shift in IC_50_. The ratio of IC_50_ values measured via high throughput method (hDHFR/tgDHFR) to the ratio measured from lysates (rat liver DHFR/TgDHFR) under different conditions are 4.6 versus 3.4, which are comparable.

Overall, JPC-2067-B has considerable potency and some selectivity relative to mammalian reference enzymes, in two independent laboratories under slightly different assay conditions demonstrating the effect of this compound on *T. gondii* DHFR.

### Molecular modeling of *T. gondii* DHFR [51] and JPC-2067-B

**Figure 6:**
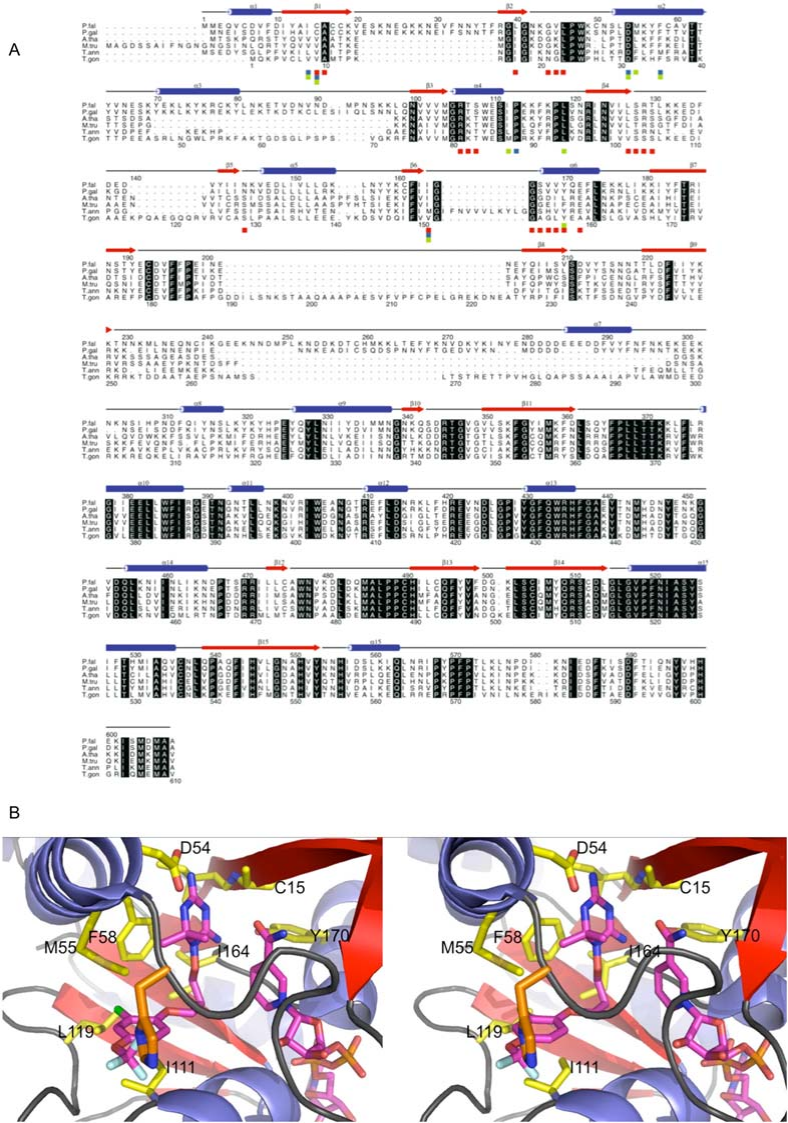
Structure based sequence alighnment of DHFR-TS and stereo view of DHFR/NADPH/WR99210 complex inhibitor binding site. A. A structure based sequence alignment of the DHFR-TS enzymes from *P. falciparum* (P. fal), *P. gallinaceum* (P. gal), *A. thaliana* (A. tha), *Medicago truncatula* (M. tru), *Theileria annulata* (T. ann) and *T. gondii* (T. gon). The sequence numbering for the *P. falciparum* and *T. gondii* is given above and below the alignment, respectively. The secondary structure elements for *P. falciparum* DHFR-TS are given above the alignment with blue cylinders and red arrows representing α-helices and β-sheets, respectively. Residues which display sequence conservation across all species are highlighted by a black box with reverse type. Those residues which are involved in binding NADPH, pyrimethamine and WR99210 are highlighted by a red, blue and green box below the alignment, respectively, with those residues which bind both inhibitors and/or NADPH displayed with multiple colored boxes. B. A stereo view of the *P. falciparum* DHFR/NADPH/WR99210 complex inhibitor binding site with the closely related inhibitor JPC-2067-B modeled. Those residues which form close interactions with the inhibitor are labeled and shown in a stick format, colored yellow, red, blue and orange for carbon, oxygen, nitrogen and sulfur, respectively. The modeled JPC-2067-B inhibitor and NADPH cofactor are colored purple, blue, red, orange, cyan and green for carbon, nitrogen, oxygen, phosphorous, fluorine and chlorine, respectively. In addition His27, in *T. gondii* DHFR, which replaces Cys50 in *P. falciparum* DHFR, is also shown in a stick format (colored orange for carbon and blue for nitrogen) to demonstrate its close proximity to the modeled JPC-2067-B inhibitor.

In order to further investigate the efficacy of the dihydrotriazines on *P. falciparum* versus *T. gondii* with regard to drug design and molecular mode of action, we have analyzed structural models of the DHFR enzyme in the apicomplexan parasites. Of the 9 residues which form interactions with the dihydrotriazine inhibitor, WR99210 in the structure of *P. falciparum* DHFR Ile14, Cys15, Asp54, Met55, Phe58, Ile111, Leu119, Ile164 and Tyr170 all are either identical or very similar in *T. gondii* DHFR (Figure 6A). In particular Asp54 and Tyr170 which make important H-bonds to the inhibitor are conserved in *T. gondii* DHFR. Furthermore, modeling studies suggest that the substitution of Ile in *P. falciparum* DHFR for Met and Val at positions 111 and 164, respectively, in *T. gondii* DHFR results in little change in the Van der Waals packing interactions made to the inhibitor (Figure 6B). Modeling of the potent inhibitor JPC-2067-B into *T. gondii* DHFR reveals that the additional trifluoromethoxy group is positioned such that it is exposed to the solvent and as such can probably be tolerated by the enzyme with respect to inhibitor binding. In addition, Cys50, which when mutated has been shown to play a role in pyrimethamine resistance [52] in *P. falciparum* DHFR, is replaced by His27 in *T. gondii* DHFR. Given its position close to the trifluoromethoxy group of JPC-2067-B it may well be that further modification to this part of the inhibitor could lead to favorable interactions with the imidazole ring of His27 (Figure 6B). However, these small changes in the *T. gondii* JPC-2067-B binding site, when compared to its homologue in *P. falciparum* may contribute to the somewhat lower sensitivity of this enzyme to JPC-2067-B.

### DHFR sequences from clonal type I, II and III parasites and atypical parasites including those found in developing tropical countries

The deduced amino acid sequences of DHFRs [43] in the data base for RH(U.S., type I), Me49 (U.S., type II), VEG (U.S., type III), and Coug (atypical), and identified by PCR of DHFRs from strains isolated from Brazil, Canada, Guyana, Guatemala, China and Sri Lanka (Table 3, [45]–[50]) were identical (data not shown).

**Table 3:** Parasites from Brazil, Guyana, Guatemala, Canada, China and Sri Lanka that were sources of DHFR analyzed.

Isolate	Country	Source	SAG2	Reference: author(date), Reference number
TgCkGa1	Guatemala	Chicken2	III,nd	Dubey et al. (2005) [50]
TgCkBr125	Brazil	Chicken30	III,nd	Dubey et al. (2006) [49]
TgCtPRC7	China	Cat21	II,nc	Dubey et al. (2007a) [48]
TgCkGy2	Guyana	Chicken4	III,nc	Dubey et al. (2007b) [46]
TgRcCa2	Canada	Raccoon3	II,nc	Dubey et al. (2008) [45]
TgDgSl12	SriLanka	Dog31	II,nc	Dubey et al. (2007c) [47]

nc = non clonal, 10 markers were used and data are in references numbers; the 10 nuclear markers include SAG1, SAG2, SAG3, BTUB, GRA6, c22-8, c29-2, L358, PK1 and a new SAG2, and an apicoplast marker Apico.

nd = only SAGII data.

### Toxicology studies

A 42-day toxicology study in CD-1 mice (Table 4) produced no histopathology findings at doses up to 98 mg/kg and no gross pathology with the exception of a reduction in the rate of weight gain. A comparable 42-day toxicology study in *Macaca fascicularis* (Table 4) produced histopathology findings at 15 mg/kg with sproradic episodes of loose stools/diarrhea that resolved upon drug withdrawal. No histopathology or gastrointestinal effects were observed over the 42-day period. 7.5 mg/kg was established as the NOAEL (No Observed Adverse Effect Level). When JPC-2056 and JPC-2067 were assayed in the Ames Test, with and without microsomal activation, no activity was exhibited with tester strains TA97, TA98, TA100, TA102 and TA1535 (Table 4).

**Table 4:** Structural and Therapeutic Comparisons of JPC-2067, JPC-2056, WR99210 and PS-15.

	JPC-2067	JPC-2056	WR99210
**Structural Class**	Dihydrotriazine	Biguanide	Dihydrotriazine
**Manufacturing Liabilities**	No	No	Yes
**TEST SYSTEM**	**TOXICOLOGICAL FINDINGS**		
**^1^Ames w/o Activation**	Not Active*	Not Active*	Not Active#
**^1^Ames with Activation**	Not Active*	Not Active*	Not Active#
**^1^CD-1 Mice**		Inhibition of Weight Gain at 98 mg/kg for 42 days	
**^1^Cynomolgous Monkey**		Intermittent Loose Stools/Diarrhea 15 mg/kg for 42 days	
**^4^Oral LD50 Rat**			1,980 mg/kg
**^4^Oral LD50 Mouse**			3,510 mg/kg
**^1^Thompson Assay LD50**		256 mg/kg @ Day 6	>128 mg/kg @ Day 6

*: Tester Strains TA97, TA98, TA100, TA102 and TA1535.

# Tester Strains TA98, TA100, TA1535, TA1537 and TA1538.

## Discussion

Our studies demonstrate that JPC-2067-B is effective against *T. gondii in vitro* with an IC50 of 20 nM and *in vivo* when administered by i.p. injection and the pro-drug JPC-2056 is effective *in vivo* when administered orally. Each of our results described herein with this novel new class of anti-folate compound, dihydrotriazine, parallels earlier findings with progenitors of this class which were not as suitable for use for humans, e.g. proguanil [12] and WR99210 [13]. The major and compelling advantages of JPC-2056, which is moving into clinical trials, is in the reduction of toxicity and development of a much more readily bioavailable compound than WR99210. WR99210 will never be a medicine for humans because of difficulties in those areas, also reflected in the effect on the mammalian enzyme, Table 1. The advantages of bioavailability, high potency, specificity, selectivity and potential for elimination of toxicities that occur with pyrimethamine either used alone or in conjunction with sulfadiazine and other medicines and because JPC-2056 will be entering clinical trials for the treatment of malaria, testing of this new class of anti-folates against the related apicomplexan *T. gondii* , was very important. Our results suggest that the activity against *T. gondii* is significant and that JPC-2056 has the potential to replace the combination of pyrimethamine plus sulfadiazine or second line drugs in the treatment of toxoplasmosis.

The modeling of *T. gondii* DHFR in complex with this family of inhibitors gives us understanding at the molecular level of why compounds of this class are highly active against *T. gondii* tachyzoites. JPC-2056 already has been optimized for pharmacokinetics and lack of toxicity and is being progressed to the clinic as a potentially effective treatment for both *P. falciparum* and *P. vivax* malaria. This ongoing work with malaria treatment provides a major benefit for the development of JPC-2056 for the treatment of toxoplasmosis.

It was of importance to determine whether in DHFR the amino acids that bind this novel, highly active triazine vary in any of the atypical parasites. Analysis of available DHFR sequences in the data base for *T. gondii* isolates called RH, Me 49, VEG and Coug parasites, i.e., from a clonal types I, II and III and atypical strains, and analyses of isolates from Brazil, Guyana, Guatemala, Canada, China, and Sri Lanka [45]–[50] demonstrates that the key amino acids for binding the triazine are conserved (data not shown). There are parasites that are genetically different in different countries, e.g. in Brazil there are a variety of genetically different parasites of clonal type I/III background with an association with a very high prevalence of retinal disease; in Northern Coastal South America highly virulent parasites that have recently been lethal or caused severe illness and death in French soldiers in French Guiana and in a recent epidemic in a village in Suriname [53]; and atypical parasites in Central America and Mexico. In Asia there are unique genotypes which differ from the typical I, II, III genotypes, and in Africa there are all the genetic clonal types of parasites. In Europe and Poland the predominant type is clonal type II, and in the U.S. there are other types but a recent abstract described predomininance of type II parasites. In an epidemic in Sea Otters in Moro Bay California and on Vancouver Island, the parasites are also atypical. Each of these parasites might have different growth rates (new isolates often grow more slowly than laboratory adapted strains, JP Dubey, personal observations) and DHFRs with slightly different sequences or significant mutations are a possibility. To begin to address this issue as it is relevant to toxoplasmosis in the developing world, we have compared the sequences of DHFRs in each of the conventional parasite clonal types (I, II, and III) from the data base, and by PCR of DHFR from isolates including a Brazilian strain, a strain from Guyana, a strain from Guatemala, a strain from Canada, a strain from China and a strain from Sri Lanka (Table 3; [45]–[50]). There are no differences in amino acid sequence of the DHFRs.

As shown in the enzyme inhibition and parasite inhibition assays herein, upon conversion of JPC-2056 to JPC-2067-B by cytochrome p 450, the product, JPC-2067-B, becomes a highly effective treatment for apicomplexan infections. Toxicological data (Table 4) supports the advancement of JPC-2056 to clinical development. A 42-day toxicology study in CD-1 mice produced no histopathology finings at does up to 98 mg/kg and no gross pathology with the exception of a reduction in the rate of weight gain. A comparable 42-day toxicology study in *Macaca fascicularis* produced histopathology findings at 15 mg/kg with sproratic episodes of loose stools/diarrhea that resolved upon drug withdrawal. Antimicrobial activities of JPC-2067 suggest that the gastrointestinal events may be related to disruptions in intestinal flora. No histopathology or gastrointestinal effects were observed over the 42-day period. 7.5 mg/kg was established as the NOAEL (No Observed Adverse Effect Level). JPC-2056 and JPC-2067 were assayed in the Ames Test with and without microsomal activation. No activity was exhibited with tester strains TA97, TA98, TA100, TA102 and TA1535. In summary, JPC-2056 has two advantages over WR99210. Biguanides are better absorbed and less toxic than their dihydrotriazine metabolites as has been well established in the case of Proguanil. Cycloguanil, the active dihydrotriazine metabolite of biguanide prodrug Proguanil is poorly absorbed and is locally toxic. In addition, WR99210 and its biguanide PS-15 possess a 2,4,5-trichlorophenoxy structural feature, which is synthesized from 2,4,5-trichloro-phenol. This phenol has the potential to generate the highly regulated toxin, 2,3,7,8-tetrachlorodibenzo-*p*-dioxin (TCDD). This issue precluded the development of both WR99210 as well as its prodrug PS-15. Significantly, JPC-2056 and its active metabolite JPC-2067-B are devoid of this liability and as such offer a significant advance in this therapeutic class (Table 4). Finally, the LD50 value for JPC-2056 at high doses in the Thompson Antimalarial Assay provides sufficient therapeutic index to justify continued clinical development.

Improved, simpler to use, less toxic drugs that are easily affordable, which can be prepared in stable solution, are needed to treat toxoplasmosis. This new biguanide, moving into clinical trials, promises to be a major advance for the treatment of those with all forms of toxoplasmosis throughout the world. The development of JPC-2056 addresses factors limiting use of current medicines in the developing world for this neglected tropical disease including ease of administration, lack of toxicity, ease of monitoring, the potential for low cost, pediatric and parenteral formulations of a new and improved medicine. This therapeutic is likely to be of special benefit for those with this neglected tropical disease in developing countries.

JPC-2067-B and JPC-2056 have considerable promise as a new class of anti-folate medicines to provide improved and less toxic means to treat toxoplasmosis as well as malaria caused by *P. falciparum* and *P.vivax* and thus to become a new standard of care for treating these diseases. The potential of these compounds to act in the absence of sulfadiazine or in conjunction with other anti-microbials such as atovaquone presents the possibility of increasing tolerance and decreasing detrimental side effects including hypersensitivity.
